# Significant influencing factors and practical solutions in improvement of clinical nursing services: a Delphi study

**DOI:** 10.1186/s12913-019-4781-y

**Published:** 2019-12-31

**Authors:** Sarieh Poortaghi, Abbas Ebadi, Mahvash Salsali, Afsaneh Raiesifar, Nayyereh Davoudi, Nima Pourgholamamiji

**Affiliations:** 1 Department of Community Health Nursing, School of Nursing and Midwifery, Tehran University of Medical Sciences, Tehran, Iran; 2 Behavioral Sciences Research Center, Life Style Institute, Baqiyatallah University of Medical Sciences, Tehran, Iran; 3 Nursing Faculty, Baqiyatallah University of Medical Sciences, Tehran, Iran; 4 School of Nursing and Midwifery, Tehran University of Medical Sciences, Tehran, Iran; 5 School of Nursing and Midwifery, Ilam University of Medical Sciences, Ilam, Iran; 6 Clinical Research Develpment Unit, Mostafa Khomeini Hospital, Ilam University of Medical Sciences, Ilam, Iran; 7 Nursing and Midwifery Care Research Center, Mashhad University of Medical Sciences, Mashhad, Iran; 8 Nursing Care Research Center (NCRC), School of Nursing and Midwifery, Iran University of Medical Sciences, Tehran, Iran

**Keywords:** Accreditation, Quality improvement, Delphi study, Nursing practice, Clinical services

## Abstract

**Background:**

Clinical services evaluation with specific indicators are very helpful to identify improvable points. This study was conducted to analyze the factors affecting the quality of clinical nursing services and offer practical solutions for accreditation of clinical nursing services.

**Methods:**

The present study was conducted using Delphi method with two rounds. At the beginning of the study a questionnaire was prepared using results of another project (clinical nursing services audit). This questionnaire was sent to 47 nursing and accreditation professionals. After the first round, causes and solutions were categorized. Then participants were asked to comment on the significance of each strategy on the prepared questionnaire.

**Results:**

In the first round of Delphi in response to the question about the main causes and solutions of low quality of nursing clinical services, 394 causes and 212 solutions were mentioned by the participants. In the second round, considering moralists and specialization in the selection of nursing managers, staffing according to workload and attendance in comprehensive exam after graduation and before entering clinical fields attained the most importance.

**Conclusion:**

Mismatch of human resources with workload and lack of clarity with regard to duties have maximum correlation with poor quality of care. Organizational structure and communication program categories gained the highest and lowest importance respectively. This information could be used by nursing managers and policy makers to plan programs in order to improve the quality of clinical nursing services.

## Background

Today, addressing the quality of services in all fields is among the fundamental priorities in leading organizations. In the health sector, in terms of services coupled with dealing with the health and lives, quality improvement and assurance have attracted increasing attention. Health care quality has diverse dimensions, definitions and interpretations [1]. One of the latest authentic definitions of health care quality defines health care quality as the extent to which health services provided to individuals and patient populations improve desired health outcomes in the community in accordance with up to date clinical knowledge [2].

The increased demand for delivering right, proper and effective health care, need for standardization and control of differences and divergences, necessity for application of ideas in the field of quality control and standardization, desire to introduce the organization and strive for excellence and ethical considerations are important factors for quality improvement in health sector services [3–5].

Nurses, as the largest group of health care providers play a key role in the continuity and quality of care and health promotion at different levels of health system [6]. The quality of nursing care is not a new concept. In fact, the quality was affected by the efforts of Florence Nightingale to regulate nursing as a specialized science and profession in public health [7]. Therefore, establishment of nursing as a practical disicipline to help patients achieve positive health care outcomes is crucial [8]. Low quality of the clinical nursing services is accompanied with poor patient satisfaction, increased morbidity and mortality, extended length of stay, increased cost per discharge, lower revenue per bed, loss of productivity, loss of reputation, litigation risks, and costs [9–11] as well as adverse outcomes for patients [9]. The continuation of scientific advances in different levels requires that nurses evaluate the care program to solve many problems among clients by integrating their technical skills and professional knowledge and, based on scientific evidence, identify patients’ problems [12]. Also, nurses have a major role in health care quality provided to clients, therefore quality improvement in nursing is the most important issue among the factors associated with the growth and survival of a health care center [13].

To date, a variety of methods and tools have been used to improve the health care quality in different countries. Among these methods, clinical governance was introduced for the first time in 1998 by the National Health Service (*which is the publicly funded national healthcare system for England and largest single-payer healthcare system in the world*) as a strategy provided by the state to enhance the quality of clinical care. Clinical governance is a framework in which organizations providing clinical services which are accountable for continuing quality improvement, are compelled to create an environment for excellence in clinical services as well as promote higher standards of service [14].

Clinical governance is considered as a framework for improving the quality of clinical services in nursing. Professional nursing practice standards are valid expression tasks that are expected of all nurses regardless of their roles and expertise in communities to be done with merit. Published standards are considered as evidence for the standard of care with the understanding that the standard application is totally dependent on the context. Due to the dynamic nature of nursing, care standards are constantly changing and being updated [9]. Compliance with these standards can be used as a tool for measuring the quality of nursing care. Clinical governance focuses on continuous improvement of the quality of clinical nursing services. Indicators selection is a major part of the quality improvement program. These indicators are written to reflect current performance and expectations for specific nursing performance [15].

Davis and colleagues indicated that measuring the health care quality provided by nurses in the health settings is a necessity [16]. Lee also indicated that in fact, the first and most important factor in improving the health care quality, is measuring it [17]. In this regard, Mrayyan’s study showed that 50% of the level of care was “very good” and 37.5% was “satisfactory”. In this study, with considering the multidimensional nature of the quality of care, the authors emphasized the role of nursing managers in improving the quality and promoting the level of job satisfaction as well as the independence of nurses in providing care [18]. Therefore evaluation with the specific indicators is exceedingly helpful to identify points that can be improved and upgraded. Finally, by proposing strategies for quality improvement in improvable points, the goal of continuous quality improvement will be achieved. For this purpose, based on assessment results from a larger study that was conducted in the form of a PhD dissertation [19], this study was conducted to analyze the significant factors affecting the quality of clinical nursing services and to offer practical solutions for accreditation of clinical nursing services using Delphi method.

## Methods

### Design

According to the objectives, this study was conducted using the Delphi method to analyze the significant influencing factors and provide practical solutions in improvement of clinical nursing services. Delphi is a systematic research approach or method for extracting opinions from a group of experts about an issue or question [20] or to reach a group consensus through a series of questionnaire rounds, considering respondents anonymity and feedback opinions to members of the panel [21]. In this study, there was a need for obtaining opinions of specialists in both Nursing and Accreditation fields and because of their lack of time and geographical distribution, it was necessary to use electronic Delphi. Decisions on the number of rounds to a great extent is practical or experiential, depending on the available time and the first question [22, 23]. Studies have been reported on the number of rounds, two to ten [24]. This survey has been conducted in two rounds as the goal was reached by doing these two rounds. Due to the ease of access to people through e-mail, this method was used to collect data on both rounds.

### Participants

Given that Delphi focuses on the extraction opinions from experts in a short time, the results depend on the expertise in terms of knowledge, quality and accuracy of answers, as well as cooperation and continued involvement in the study [25]. In this study, both nursing (*n* = 29) and accreditation professionals (*n* = 18) were identified and involved in the study through purposive sampling (Table 1). In order to achieve maximum variation, the researchers tried to select participants based on variation of education and expertise levels.

**Table 1:** Demographic characteristics of the study participants

Demographic characteristics		Frequency	Percentage	Total
Gender	Male	33	70.21	100
	Female	14	29.79	
Educational level	PhD	28	59.79	100
	MSc	8	17.02	
	BSc	11	23.41	
Specialization	Accreditation Officer	14	29.79	100
	Nurse Manager	14	29.79	
	Clinical nurse educator	5	10.64	
	Head of quality improvement office	4	8.51	
	Clinical nurse	10	21.27	
Years of experience	5–10 years	7	14.89	100
	10–15 years	21	44.68	
	> 15 years	19	40.43	

Moreover, as the first author has extensive experience in both clinical and quality assurance fields and she is the national hospital accreditation assessor of the Ministry of Health, she is familiar with experts in this field. Identification of experts took place with regard to inclusion criteria such as experience in the field of accreditation or nursing Management, at least 5 years of relevant work experience, availability and satisfaction to participate in the panel. Experts from various settings including twenty governmental hospitals, eight private hospitals, three faculties as well as the Ministry of Health were enrolled in the study.

### Procedure

Initially, in the form of an email that was followed by a phone call, the goals of the study were explained to the participants by the researcher and informed consent was received for participation in the study. In the first round, using the results of clinical nursing services aduit which was conducted by a valid and reliable checklist on 300 nurses working in hospitals affiliated to Tehran University of Medical Sciences and is described in another article [19], a questionnaire was prepared with 18 indicators. These indicators were taken in auditing score below 70 and were intended as abnormal according to the national hospital accreditation cut off point (Iranian Hospital accreditation audit guide, 2013). The questionnaire was sent along with the cover letter including information such as: introduction of the purpose of the research and an explanation of why he /she has been chosen for the panel, description of the initial phase of the study and how to extract unfavorable indicators, full explanation of how to complete and return the questionnaire, emphasis on the privacy of experts and their comments as well as how to contact the researchers in case of necessity (Additional file 1). The experts were asked to mention the possible causes of low scores in front of each indicator in the first column, and strategies for improving scores and thus improving the quality of clinical nursing services in the second column. After collecting the questionnaires, causes and solutions were categorized using content based method. In this way, the different causes mentioned by participants against each indicator were merged, repeated cases removed and causes that were more comprehensive and covered the larger number of cases were retained. The collected solutions were analyzed firstly in terms of their repetition and more comprehensive sentences that included several solutions were developed. Then remaining solutions were categorized based on their similarities.

In the second round, to achieve the aim of providing practical solutions to improve the quality of clinical nursing services, based on data from the first round, a questionnaire was designed. The questionnaire consisted of categorized solutions (37 solutions) on 8 categories. Participants were asked to comment on the significance of each of these strategies in order to improve the quality of clinical nursing services in the five-item Likert scale (Additional file 2).

The deadline for returning the questionnaires of each of the two Delphi rounds was considered 1 week in the cover letter of the questionnaire. During this time, a reminder email was sent twice. In addition to raising the number of returned questionnaires, the period of waiting was considered 3 days more than the deadline (10 days in total).

### Data analysis

In the first round of Delphi, all collected data were entered in Open Code software [version 4.2] and were analyzed using conventional content analysis. At the first, codes were extracted from data inductively and coding list was prepared (212 solutions (codes) were provided). At this phase all codes and their relevent explanation were provided. After the codes have been identified, the original expert responses was re-read alongside the final list of codes. This step was performed due to ensure that all aspect of experts’ opinion were covered in terms of study aims. Then sub-categories and categories was developed according to codes similarities. At this step the extracted codes were classified in 37 solutions (sub-categories). Finally, by further comparison, these solutions were merged into 8 categories. In the second-round, a questionnaire was designed in which classified solutions (37 solutions) were listed and the participants were asked to comment on the significance of each of these strategies in the five-item Likert scale in order to improve the quality of clinical nursing services. Likert scale was considered as 5 points: absolutely important (score 5), important (score 4), moderately important (score 3), a little important (score 2) and not at all important (score 1). The collected data in the second phase of the study were analyzed by SPSS V/16 and using descriptive statistical methods (mean, standard deviation, and frequency).

## Results

Most of the participants were male (*n* = 33 (70.21%)), with a PhD degree of education (*n* = 28 (29.79%)) and work experience of 10–15 years (*n* = 21 (44.68%)). Demographic characteristics of the participants involved in the current study have been shown in Table 1.

In the first round of Delphi, from a total of 46 questionnaires, 38 questionnaires (60.82%) were returned. In this round, 394 causes and 212 solutions were offered. After collecting the questionnaires, causes and solutions were categorized. Table 2 shows influencing factors which are related with low scores of indicators. As it was shown, mismatch of nursing human resources with workload and lack of clarity of the nurses’ duties have maximum correlation with poor quality of nursing care.

**Table 2:** Influencing factors of nursing clinical services accreditation

No	Indicator with low score	Influencing factors
1	Records care plan in a way that is valid (POR / POMR / HIS), understandable and accessible by all members of the health care team	1. Lack of sufficient knowledge and skills of nurses 2. Mismatch of nursing human resources with workload 3. Lack of care plan documentation software 4. Lack of proper supervision 5. Lack of consensus on the necessity of delivering care according to a care plan 6. Failure to update documentation guidelines in nursing
2	Cooperates with other health care team members in line with the care plan	1. Mismatch of nursing human resources with workload 2. Lack of familiarity with the capabilities of other health care team members 3. Lack of attention to holistic care 4. Lack of team work culture in all health care team members 5. Lack of organizational communication protocols
3	Before doing clinical interventions, expertise of nurse has been proven (specialized courses have been completed)	1. Lack of special professional bodies for evaluation and licensing expertise of nurses (registered nurse program) 2. Extensiveness of nursing interventions and tasks 3. Limited use of performance tests such as the OSCE for evaluation of students and nursing staff 4. Failure to update and development of short-term and long-term in-service training 5. Failure to investigate nurses competencies at the time of recruitment 6. Low motivation of nurses for participating in necessary in-service courses
4	In case of doubt in any action or intervention gets help from skilled, competent and capable colleagues	1. Feel the inability 2. Assume insignificant to safe care 3. Fear of humiliation and punishment 4. Lack of teamwork culture 5. Lack of motivation in educational mentors to train the new colleuges 6. Distrust of the knowledge and skills of colleagues and other scientific groups in the field 7. The absence of protocols and manuals for similar situations 8. Lack of appropriate regulatory measures 9. Working routine-based and not performing interventions in the systematic and scientific form
5	Introduces him/her self to the patient and his family in the first encounter	1. Insufficient attention to patient rights and respect of clients 2. Culture of patient rights 3. Inappropriate indigenous culture 4. Mismatch between nursing human resources with workload 5. Focus on the disease instead of the patient 6. Lack of attention to professional ethics codes 7. Lack of protocols for communicating with patients 8. Lack of nurse qualifications assessment at the time of employment
6	Ensures that patients and families have access to a support group in the community and for continuously improving patient’s condition, actively seeks feedback from patients and his family	1. Nurses work load and the lack of time to track 2. The lack of holistic care systems and community-based programs in nursing of Iran 3. Lack of clarity of the duties 4. Inadequate supervision 5. The lack of a suitable platform to provide nursing supportive role
7	Has adequate, timely and fair attention to the patient’s complaints and demands	1. Weakness in the follow-up, referral and resolving complaints system 2. Mismatch of nursing human resources with workload 3. Lack of clarity of the duties 4. Lack of necessary professional commitment and in some cases conflict of professional commitment with an organizational commitment 5. Lack of knowledge and attitude of nurses about their supporting role 6. Lack of patient-centered care 7. Lack of awareness of the complaints process
8	Investigation of any possibility of pressure and damage to the skin caused by sedentary position and taking the necessary interventions to prevent damage to skin	1. Lack of interest in the nursing profession and patient care 2. Mismatch of nursing human resources with workload 3. Lack of clarity of the duties 4. Neglect and lack of responsibility 5. Unidimensionality and routine care 6. Having technical view to nursing services instead of professional view 7. Traditional management of nursing services and using functional method of care delivery instead of patient-centered practices (case method, etc.) 8. Low knowledge and skills of nurses 9. The absence of control and supervision
9	Cooperates with the patient and family in decision making and care dimensions which are related to mobility and patient safety as far as possible	1.Lack of comprehensive, patient-centered programs in hospital 2. Lack of effective communication with patient’s family 3. Mismatch of nursing human resources with workload 4. The lack of awareness of patients about their rights in terms of participatory decision-making 5. Lack of attention to the importance of the issue 6. Prevailing culture of paternalism
10	At the time of admission, assesses the patient’s nutritional needs and identifies any current or potential problem	1. Lack of awareness about the importance of the issue 2. Weakness in medical history-taking 3. Unclarity of the duties 4. Inadequate nutritional information of head nurses and clinical educators 5. Lack of training in this area due to nursing shortage 6. Mismatch of nursing human resources with workload 8. Lack of patient/family centered care
11	Helps patients who cannot eat	1. Lack of clarity of the duties 2. Giving low importance to nutritional needs 3. Mismatch of nursing human resources with workload 4. Inadequate supervision
12	To maintain good health, encourages patients to eat and drink	1. Lack of clarity of the duties 2. Lack of awareness of the necessity of issue 3. Inadequate supervision 4. Insufficient emphasis on this issue during the course 5. Inattention to patient needs 6. Mismatch of nursing human resources with workload
13	Monitors food and fluid intake and Records if necessary	1. Mismatch of nursing human resources with workload 2. Lack of awareness of the necessity of issue 3. Insufficient emphasis on this issue during the course 4. Inadequate supervision
14	Records interventions taken and outcomes related to patient’s nutritional inadequacy	1. Insufficient emphasis on this issue during the course 2. Mismatch of nursing human resources with workload 3. Lack of awareness of the necessity of issue 4. Lack of clarity of the duties
15	Evaluates care or assistance needed to maintain and improve personal hygiene (dental, skin and hair, ...) on admission of the patient	1. Deficiency of comprehensive view on admission 2. Lack of awareness of the necessity of the issue 3. Inadequate supervision and education 4. Mismatch of nursing human resources with workload
16	Records personal hygiene outcomes correctly and in accordance with the relevant policies and procedures	1. Mismatch of nursing human resources with workload 2. Policies are not formulated clearly, notification or monitoring is not enough 3. Lack of motivation for patient care 4. Inadequate supervision and control
17	Evaluates patient on admission, identifies and records any psychological or physical disability that is likely to be dangerous for him	1. Emphasis on the completion of the written work and the legal description of the duties and undermining the physical and emotional issues 2. Lack of a comprehensive, operational and applicable protocols for patient admission 3. Unfamiliarity with the need for a holistic and humanistic look to the patients 4. Lack of collection and monitoring system as well as root causes analysis 5. Mismatch of nursing human resources with workload 6. Lack of awareness of the necessity of issue 7. Inadequate education 8. Inadequate supervision and control
18	Records any risk analysis and the actions needed to reduce / eliminate the risk	1. Lack of clarity of documentation protocol for recording risk factors 2. Deficiency of knowledge, skill, and regulational awareness regarding risk management 3. Lack of proper supervision Failure to understand the importance of risk management 4. Nursing shortage

**Fig. 1:**
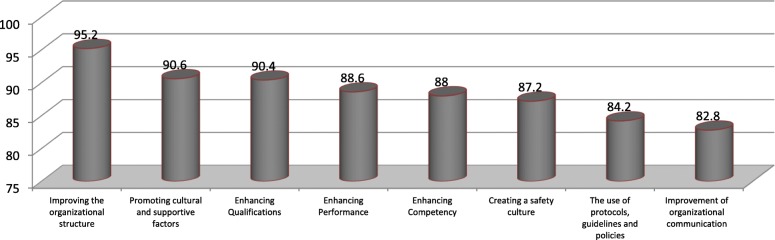
Practical strategies for improving the quality of clinical nursing services in order of importance in experts’ opinions

From the 38 questionnaires sent, 32 pcs (84.21%) were returned. Data analysis in this phase is show in Table 3. As it was revealed, attention to morality and specialization in selection of nursing managers (96.2%) and then providing nursing staff according to the workload (95%), comprehensive exams before entering the clinic (95%) received the most importance among the proposed solutions by experts. Fig. 1 shows practical strategies for improving the quality of clinical nursing services in order of importance in the experts’ opinions. As can be seen, Improving the organizational structure achieved the highest and Improvement of organizational communication the lowest rank in the opinion of the experts in this study.

**Table 3:** Practical solutions to improve the quality of clinical nursing services

Solution	Significance	
	Mean (SD)	Frequency (Percent)
Category 1: Enhancing Qualifications		
1. Revise the way of attracting students	4.62 (0.83)	92.4%
2. Revision of nursing education curriculum	4.15 (0.80)	83%
3. Comprehensive exam	4.75 (0.50)	95%
4. Conducting registered nurse program	4.53 (0.76)	90.6%
5. Strengthen strategies to absorb nurses	4.40 (0.72)	88%
Mean	4.52 (0.33)	90.4%
Category 2: Enhancing Competency		
1. Empowering of nursing staff	4.53 (0.56)	90.6%
2. Short courses in accordance with the new requirements	4.28 (0.81)	85.6%
3. Change the type of assessments	4.37 (0.60)	87.4%
4. Change the organizational climate	4.56 (0.54)	93%
5. More serious attention to mentorship method	4.18 (0.78)	83.6%
Mean	4.40 (0.46)	88%
Category 3: Improvement of organizational communication		
1. Develop communication policy and procedure	3.96 (0.73)	79.2%
2. Create a culture of teamwork	4.46 (0.50)	89.2%
3. Joint educational and research training courses	4.09 (0.73)	81.8%
4. Modification of the method of work shifts change	4.06 (0.71)	81.2%
Mean	4.14 (0.46)	82.8%
Category 4: Enhancing Performance		
1. Strengthen the holistic care	4.53 (0.46)	90.6%
2. Emphasis on the elimination of irrelevant duties	4.46 (0.71)	89.2%
3. Strengthen patient education	4.31 (0.69)	86.2%
Mean	4.13 (0.43)	88.6%
Category 5: The use of protocols, guidelines and policies		
1. Update record and report instructions in nursing	4.40 (0.71)	88%
2. Provide a valid international checklist to assess, prevent and improve complications of treatment	4.12 (0.83)	82.4%
3. Create protocols on patient assessment	4.15 (1.01)	83%
4. Create policy for patients decision making and self-care	4.34 (0.78)	86.8%
5. Create assessment tools for monitoring documents	4.15 (0.76)	83%
6. Design of applicable protocols for patient admission	4.09 (0.92)	81.8%
Mean	4.21 (0.59)	84.2%
Category 6: Creating a safety culture		
1. Enhancing skills of nursing staff regarding risk management and patient safety	4.18 (0.64)	83.6%
2. Creating a culture of medical errors reporting	4.53 (0.62)	90.6%
3. Designing an operational protocol for risk recording	4.37 (0.94)	87.4%
Mean	4.36 (0.51)	87.2%
Category 7: Promoting cultural and supportive factors		
1. Enhancing motivation among nurses	4.68 (0.59)	93.6%
2. Improving knowledge and attitudes of nursing managers to provide professional nursing services	4.65 (0.48)	93%
3. Holistic View health system strengthening	4.59 (0.49)	91.8%
4. Creating an appropriate context for the supportive role of nurses	4.43 (0.61)	88.6%
5. Strengthening the patient-centered culture	4.31 (0.85)	82.6%
6. Infrastructuring for patients rights as well as follow-up process of created problems	4.56 (0.71)	91.2%
7. Real implementation of the provisions of the Charter of Patients’ Rights	4.43 (0.71)	88.6%
8. Applying professional codes of ethics	4.56 (0.61)	91.2%
Mean	4.53 (0.42)	90.6%
Category 8: Improving the organizational structure		
1. Efficient and continious monitoring system for nursing	4.71 (0.63)	94.2%
2. Staffing according to workload	4.75 (0.43)	95%
3. Moralists and specialization in the selection of nursing managers	4.81 (0.38)	96.2%
Mean	4.76 (0.30)	95.2%

## Discussion

In this study, Delphi method was used in order to achieve the objectives of the analysis for the factors affecting the quality of clinical nursing services and to provide practical solutions to accreditation of clinical nursing services.

The results of this study showed that mismatch of nursing human resources with workload and lack of clarity of the nurses’ duties have maximum correlation with poor quality of nursing care according to experts’ opinions. These factors lead to dissatisfaction in the nurses. In a study by Aiken et al., dissatisfaction and burnout were the most common problems in all five study regions and two variables of organizational support and human resources in nursing, which had an independent and direct realtionship with quality of care. They stated that sufficient human resources as well as organizational and managerial support are key to improving the quality of patient care and reducing dissatisfaction and burnout occurrances [26]. These findings are in line with the findings of this study. While nursing interventions are closely related to the patient’s life, the quality of care is largely dependent on the proper supply of skilled nursing [27]. Studies show a direct link between the availability and productivity of nurses and quality of nursing care [28]. Decreased quality of care, inadequate care of patients, increased inpatient mortality and postoperative complications, nosocomial infections, medical errors and infections in hospitals and other negative outcomes are adverse consequences of nurse shortage [29] which due to its key role in promoting clinical nursing services, should be considered by policy makers. Current health policies require the necessary process to make changes and form support groups for nurses and support the emergence of advanced nurse practitioner that seeks to eliminate barriers and support the provision of the best care for their patients.

Another cause of the low quality of care in this study was the lack of motivation to provide care. As Oshvandi et al. [27], stated, the nature of nursing duties requires that employees do their work with passion and love. Since nursing intervention is closely related to patient’s life, lack of motivation among nurses leaves adverse effects on the safety of patients. Nursing managers should try to identify effective factors in increasing nurses’ motivation and act in order to improve it, thereby improving the quality of nursing care.

The lack of appropriate regulatory measures on the performance of nurses is another problem. The allocation of sufficient resources (money, materials and manpower), dedicated time for research, audit and benchmark marketing, management support and continuous monitoring are the major infrastructure to maintaining quality in health services. Mousavi et al. [30] states that nurses often work as employees and act in accordance with the rules governing the organization, therefore it confers a profound impact on their performance. In a study Nasiriani et al. showed that clinical monitoring is one of the central activities in the caring professions and is treated as a main way for support and professional development of nurses. Clinical supervision is introduced as the second of three major leaders’ behaviors including nurse performance observation, evaluation, upgrading or modifing it, as well as a tool for quality assurance of nursing care [31]. In the study of Ravaghi et al. [32], facilitating factors for clinical monitoring, including knowledge and positive attitude towards clinical governance, supportive atmosphere, managers commitment, effective communication and effective motivation were designed and barriers included reverse listed facilitators in addition to inadequate resources, legal challenges, high workload and parallel quality control programs.

In this study, among the solutions proposed to improve the quality of clinical nursing services, moralists and specialization strategies (adequate management expertise) in the appointment of nursing and non-nursing administrators and then providing nursing staff according to the workload, comprehensive exams to determine competency before entering the clinic received the most importance from the point of view of experts. Developing communication policies and procedures in the organization when handling patient information is also allocated to least important. Mc Sherry et al. [33], have stated that excellence in nursing only occurs when the ability of managers, leaders and educators of nurses is ensured in response to the complexity of the reform and change in leadership, management, empowerment, encouraging and providing a creative and innovative human resource is obtained. This reflects the importance of nurse managers selection. Mousavi et al. [30], stated that transparency in leadership roles and their accountability are very important in clinical governance. It is obvious that the ambiguity in responsibilities, transformational leadership and long-term unclarity leads to a feeling of helplessness among the leaders and becomes an impediment to the clinical governance.

Baghaee et al., wrote 75 % of nurse managers and 64.3% of supervisors had moderate knowledge about professional nursing management principles. In addition, 50% of nurse managers and 57% of supervisors had moderate management performance. Therefore, it is necessary to improve knowledge, attitude and practice of nursing managers about professional nursing management principles to achieve optimal performance [34]. Results of Mrayyan and Acorn, showed that five important variables affecting the quality of care from the perspective of supervisors were the time to perform tasks, the number of adequate nursing staff at all levels, timely medication orders adminstration, preparing and maintaining the equipment [18]. In this study, staffing according to the workload that scored high importance from the perspective of experts is in line with our study.

## Conclusions

The strength of this study is that the basic information to guide the project was based on clinical audit of nursing services using valid and reliable checklist. In the other hand, initial data was extracted from real situations and we can rely on the results. Results of this study showed that mismatch of nursing human resources with workload and lack of clarity of the nurses’ duties have maximum correlation with poor quality of nursing care according to experts’ opinions. Furthermore, in terms of practical solutions to improve the quality of clinical nursing services, organizational structure and communication program categories gained the highest and lowest importance respectively. Continuous improvement of clinical nursing services is achievable by auditing and offering improvement programs. Creating adequate motivation among nurses is necessary for this purpose. The plan to change the existing structures in the form of solutions presented in this study can be considered as an effective step to improve the quality of clinical nursing services. The e-Delphi methodological constraints also affected this research. These limitations, including access to the internet and work with email, may affect the response rate and in this study we had low response rate. In spite of coding for participants anonymity, it was possible for the first researcher to identify them via their email address. In addition, in this study, due to time constraints as well as increasing the likelihood of attrition with the increase in the number of rounds, Delphi study was conducted in just two rounds.

## Supplementary information

**Additional file 1:** The primary questionair for the first round (factors associated with a low score on some of the indicators related to the accreditation of clinical nursing services were collected based on expert comments).

**Additional file 2:** The questionnair for the second round( the importance of each of strategies in enhancing the quality of clinical nursing services).

## Data Availability

The datasets generated and/or analysed during the current study are not publicly available due [they are confidential data and are in persian language] but are available from the corresponding author on reasonable request.

## Abbreviations

NHS: National Health System
