# Supplementary material for: Significant influencing factors and practical solutions in improvement of clinical nursing services: a Delphi study
Source: BMC Health Serv Res. 2019 Dec 31;20:3. doi: 10.1186/s12913-019-4781-y (PMC6937807; doi:10.1186/s12913-019-4781-y)
Supplement: Supplementary file 2 — Additional file 2: The questionnair for the second round( the importance of each of strategies in enhancing the quality of clinical nursing services). [file 12913_2019_4781_MOESM2_ESM.docx]

**Dear colleagues and professors**

**Greetings and respect**

**In the first round of this study, the factors associated with a low score on some of the indicators related to the accreditation of clinical nursing services were summarized. The strategies presented by the experts are presented in the table below, along with the strategies that have been extracted from the literature review and the issues that have been identified in the process of conducting the research. Please comment on the importance of each of these strategies in enhancing the quality of clinical nursing services.**

**Best regards**

| **Significance** | | | | | **Solution** |
| --- | --- | --- | --- | --- | --- |
| **Unimportant** | **Slightly Important** | **Moderately Important** | **Important** | **Very Important** |  |
|  |  |  | **Category 1: Enhancing Qualifications** | | |
|  |  |  |  |  | 1. Revise the way of attracting students |
|  |  |  |  |  | 1. Revision of nursing education curriculum |
|  |  |  |  |  | 1. Comprehensive exam |
|  |  |  |  |  | 1. Conducting registered nurse program |
|  |  |  |  |  | 1. Strengthen strategies to absorb nurses |
|  |  |  | **Category 2: Enhancing Competency** | | |
|  |  |  |  |  | 1. Empowering of nursing staff |
|  |  |  |  |  | 1. Short courses in accordance with the new requirements |
|  |  |  |  |  | 1. Change the type of assessments |
|  |  |  |  |  | 1. Change the organizational climate |
|  |  |  |  |  | 1. More serious attention to mentorship method |
|  |  |  |  | **Category 3: Improvement of organizational communication** | |
|  |  |  |  |  | 1. Develop communication policy and procedure |
|  |  |  |  |  | 1. Create a culture of teamwork |
|  |  |  |  |  | 1. Joint educational and research training courses |
|  |  |  |  |  | 1. Modification of the method of work shifts change |
|  |  |  | **Category 4: Enhancing Performance** | | |
|  |  |  |  |  | 1. Strengthen the holistic care |
|  |  |  |  |  | 1. Emphasis on the elimination of irrelevant duties |
|  |  |  |  |  | 1. Strengthen patient education |
|  |  |  | **Category 5: The use of protocols, guidelines and policies** | | |
|  |  |  |  |  | 1. Update record and report instructions in nursing |
|  |  |  |  |  | 1. Provide a valid international checklist to assess, prevent and improve complications of treatment |
|  |  |  |  |  | 1. Create protocols on patient assessment |
|  |  |  |  |  | 1. Create policy for patients decision making and self-care |
|  |  |  |  |  | 1. Create assessment tools for monitoring documents |
|  |  |  |  |  | 1. Design of applicable protocols for patient admission |
|  |  |  | **Category 6: Creating a safety culture** | | |
|  |  |  |  |  | 1. Enhancing skills of nursing staff regarding risk management and patient safety |
|  |  |  |  |  | 1. Creating a culture of medical errors reporting |
|  |  |  |  |  | 1. Designing an operational protocol for risk recording |
|  |  |  | **Category 7: Promoting cultural and supportive factors** | | |
|  |  |  |  |  | 1. Enhancing motivation among nurses |
|  |  |  |  |  | 1. Improving knowledge and attitudes of nursing managers to provide professional nursing services |
|  |  |  |  |  | 1. Holistic View health system strengthening |
|  |  |  |  |  | 1. Creating an appropriate context for the supportive role of nurses |
|  |  |  |  |  | 1. Strengthening the patient-centered culture |
|  |  |  |  |  | 1. Infrastructuring for patients rights as well as follow-up process of created problems |
|  |  |  |  |  | 1. Real implementation of the provisions of the Charter of Patients' Rights |
|  |  |  |  |  | 1. Applying professional codes of ethics |
|  |  |  | **Category 8: Improving the organizational structure** | | |
|  |  |  |  |  | 1. Efficient and continious monitoring system for nursing |
|  |  |  |  |  | 1. Staffing according to workload |
|  |  |  |  |  | 1. Moralists and specialization in the selection of nursing managers |
