# Supplementary material for: Significant influencing factors and practical solutions in improvement of clinical nursing services: a Delphi study
Source: BMC Health Serv Res. 2019 Dec 31;20:3. doi: 10.1186/s12913-019-4781-y (PMC6937807; doi:10.1186/s12913-019-4781-y)
Supplement: Supplementary file 1 — Additional file 1: The primary questionair for the first round (factors associated with a low score on some of the indicators related to the accreditation of clinical nursing services were collected based on expert comments). [file 12913_2019_4781_MOESM1_ESM.docx]

**In the name of God**

**Dear professor**

**Greetings and respect**

 I am Sarieh Poorteghi Ph.D. student of Tehran University of Medical Sciences. My PhD dissertation, entitled "Accreditation of Nursing Clinical Services: Explaining and Analyzing Effective Factors and Providing Practical Solutions", under the supervision of Prof. Salsali directed in two phases; Qualitative Phase (Developing of Accreditation Checklist for Nursing Clinical Services) and Quantitative phase ( Nursing Clinical Services Auditing, using a designed checklist). In this study, all of the nursing clinical services of 300 nurses working in hospitals affiliated to Tehran University of Medical Sciences were evaluated using 66 indicators designed in the level of individual performance evaluation. Unfortunately, in some of the indicators, there was no satisfactory score which have been listed in the following table.

It should be noted that the score below 70 is considered undesirable. Considering that in order to making the results of this study applicable, it is necessary to provide solutions to improve the quality of clinical services in the field of nursing and to use your expertise, It would be appreciated if you accompany us in this way so that we can take steps to promote the nursing profession.

Please in front of each indicator list the possible causes that may be involved in the undesirable scores of the relevant indicator and the solutions that can be used to eliminate these factors and increase the indicator score and, consequently, increase the quality of care.

It should be noted that after summarizing the comments of all the participants involved in this study, you will be sent back again to confirm the results.

**Thanks for your participation.**

| **No** | **Indicator** | **Indicator score**  **Mean (SD)** | **Possible Causes** | **Solution** |
| --- | --- | --- | --- | --- |
| 1 | Records care plan in a way that is valid (POR / POMR / HIS), understandable and accessible by all members of the health care team | 69.83(2.26) |  |  |
| 2 | Cooperates with other health care team members in line with the care plan | 67.33 (2.71 |  |  |
| 3 | Before doing clinical interventions, expertise of nurse has been proven (specialized courses have been completed) | 26.66 (2.39) |  |  |
| 4 | In case of doubt in any action or intervention gets help from skilled, competent and capable colleagues | 67.66 (2.70) |  |  |
| 5 | Introduces him/her self to the patient and his family in the first encounter | 64.16 (1.99) |  |  |
| 6 | Ensures that patients and families have access to a support group in the community and for continuously improving patient's condition, actively seeks feedback from patients and his family | 43.50 (2.27) |  |  |
| 7 | Has adequate, timely and fair attention to the patient's complaints and demands | 67.50 (1.93) |  |  |
| 8 | Investigation of any possibility of pressure and damage to the skin caused by sedentary position and taking the necessary interventions to prevent damage to skin | 52.66 (2.88) |  |  |
| 9 | Cooperates with the patient and family in decision making and care dimensions which are related to mobility and patient safety as far as possible | 55.33 (2.87) |  |  |
| 10 | At the time of admission, assesses the patient's nutritional needs and identifies any current or potential problem | 68.66 (2.07) |  |  |
| 11 | Helps patients who cannot eat | 31.66 (2.69) |  |  |
| 12 | To maintain good health, encourages patients to eat and drink | 46 (2.88) |  |  |
| 13 | Monitors food and fluid intake and Records if necessary | 48.66 (2.89) |  |  |
| 14 | Records interventions taken and outcomes related to patient's nutritional inadequacy | 55.33 (2.87) |  |  |
| 15 | Evaluates care or assistance needed to maintain and improve personal hygiene (dental, skin and hair, ...) on admission of the patient | 67.16 (1.77) |  |  |
| 16 | Records personal hygiene outcomes correctly and in accordance with the relevant policies and procedures | 65.83 (1.83) |  |  |
| 17 | Evaluates patient on admission, identifies and records any psychological or physical disability that is likely to be dangerous for him | 64.66 (1.84) |  |  |
| 18 | Records any risk analysis and the actions needed to reduce / eliminate the risk | 55 (1.95) |  |  |
